# The ultrastructure of the muscle coat of human gastro-oesophageal junction, with special reference to “interstitial cells of Cajal”^†^

**DOI:** 10.3389/fnins.2013.00049

**Published:** 2013-04-04

**Authors:** Maria-Simonetta Faussone-Pellegrini, Camillo Cortesini, Paolo Romagnoli

**Affiliations:** ^1^Department of Experimental and Clinical Medicine (Formerly: Department of Anatomy, Histology and Forensic Medicine, and in 1977 Institute of Histology and general Embryology), University of FlorenceFlorence, Italy; ^2^Chair of Experimental Surgery (in 1977; the Chair is No More Active), University of FlorenceFlorence, Italy

**Keywords:** interstitial cells of Cajal, electron microscope, gastro-oesophageal sphincter, stomach, man

## Abstract

The muscle coat of the human lower oesophageal sphincter and stomach was studied 5 cm above and 4 cm below the gastro-oesophageal junction. Four subjects were operated on for motility disorders of the esophagus, two for a hypertensive lower oesophageal sphincter and two for an epiphrenic diverticulum; six subjects were operated on for oesophageal or gastric carcinomas. Specimens were fixed in phosphate-buffered OsO_4_, embedded in Epon, contrasted with uranyl acetate and lead citrate and observed under a Siemens Elmiskop Ia electron microscope. Both the oesophageal and gastric muscle cells, which showed features typical of this cell type, were innervated by multiple varicosities that were rich in synaptic vesicles; these varicosities were generally rarely encountered at distances less than 1000 Å from muscle cells. Only a very few, close neuromuscular junctions were detected. Special cells, which correspond to the “interstitial cells of Cajal” as reported by other authors, were discerned at the periphery of muscle cell bundles. These cells were characterized by an elongated cell body with many thin branches and an oval, sometimes indented nucleus. Some pinocytotic vesicles were located at the cell periphery. These cells were surrounded by a discontinuous basal lamina and were seen in close contact with each other and with muscle cells; the close contact areas were often very wide. The cytoplasm contained variable amounts of mitochondria, a well-developed smooth endoplasmic reticulum and a Golgi complex. As a characteristic feature, bundles of thin filaments were located at the cell periphery and were attached to electron-dense areas of the cell membrane. Morphologically, these filaments resembled myofilaments; they were present in variable amounts and were sometimes very numerous. The observation that the cytoplasmic organelles and filaments varied in number, is probably related to the different functional properties of these cells. Interstitial cells were richly innervated by varicose nerve fibers that were densely packed with synaptic vesicles; many close junctions to nerve endings were also detected. These morphological data lead us to assume that the interstitial cells demonstrated by the electron microscope do not correspond to the cells initially identified by Cajal and cannot even be considered connective tissue cells. We propose that they are specialized smooth muscle cells that are involved in generating spontaneous, myogenic electrical activity in the gastrointestinal tract.

## Introduction

Due to its role in peristalsis, the muscle coat of the digestive system has long since caught the attention of investigators, resulting in a multitude of electron microscopic studies dedicated to this structure (Richardson, 1958; Yamamoto, 1960; Taxi, 1961, 1964; Harman et al., 1962; Lane and Rhodin, 1964; Oosaki and Ishii, 1964; Cassella et al., 1965; Gabella, 1967a,b, 1973; Dewey and Barr, 1968; Pellegrini, 1968a,b; Schofield, 1968; Silva et al., 1968; Silva, 1971; Faussone Pellegrini, 1972). The smooth muscle cells of the muscle coat of the digestive tract are large, spindle-shaped cells that form small bundles connected by thin septa. The elongated, centrally located nucleus has an uneven profile. The few organelles (mitochondria, cisternae of rough endoplasmic reticulum, ribosomes, and a small Golgi apparatus) are concentrated close to the nuclear poles. The cytoplasm is filled with thin myofilaments that are aligned with the major cell axis and are sometimes associated with the plasma membrane in electron-dense areas. Outside these areas, the plasma membrane is rich in pinocytotic vesicles. The uneven cell surface is caused by pointed cell projections that make epithelial cell-like contacts with neighboring cells. Thereby, plasma membranes lies close to each other, with an intervening, electron transparent-space (180–200 Å) from which the basal lamina is excluded. Overall, it can be concluded that these cells have the typical features of smooth muscle tissue cells.

Special attention has been paid by investigators to the nerve component of the muscle coat of the digestive system which is made up of small bundles of unmyelinated nerve fibers that run among the muscle cell bundles and from which thin branches depart to enter the muscle cell bundles. Axons dilate into varicosities, some of which contain small electron-transparent vesicles, while others contain large vesicles with an electron-dense content. The former vesicles are considered to be cholinergic; the latter appear to have ATP or a related nucleotide as mediator, as suggested by recent pharmacological evidence (Uehara et al., 1976). These vesicles have accordingly been designated as “purinergic” (Burnstock, 1971). The relationships between axons and muscle cells are mediated by a number of specific structures: true intercellular junctions are rare; more often synaptic vesicle-rich varicosities are more than 1000 Å away from the smooth muscle cells (Bennett and Rogers, 1967; Gabella, 1972) and nerve fibers run parallel to the smooth muscle cells over long distances while dilating into multiple varicosities.

To our knowledge, the third component and by far most elusive element of the muscle tissue of the digestive tract, i.e., the so-called “interstitial cells of Cajal,” has only been described in the intestinal muscle coat. These cells were identified by Cajal (1889, 1895, 1911) in some mammals, including humans, and have since been studied by both light and electron microscopy; their nature and functional role are still unknown. Interstitial cells of Cajal are star-like or spindle-shaped cells with long, irregularly dilated processes; they are devoid of neurofibrils and Nissl bodies and do not contact fibers of the enteric plexus. In spite of these morphological features, Cajal (1889) and several other authors (Taxi, 1952; Meyling, 1953; Honjin, 1956; Dupont and Sprinz, 1964) proposed a neural nature for these cells, whereas other authors argued that these cells are connective tissue cells (Kölliker, 1894; Dogiel, 1895, 1898; Huber, 1913; Kuntz, 1923; Johnson, 1925; Ottaviani and Cavazzana, 1940; Knoche, 1952; Weber, 1952). Interstitial cells of Cajal do not react for cholinesterase (Coupland and Holmes, 1958; Leaming and Cauna, 1961) and hence cannot belong to the cholinergic innervation system, nor has any evidence ever been found that they would be a type of neuroglia intercalated between postganglionic nerve fibers and smooth muscle cells, as proposed some time ago (Lawrentjew, 1926; van Esveld, 1928; Boeke, 1933, 1949; Schabadasch, 1934; Stöhr, 1935). Interstitial cells of Cajal are not macrophages either, as suggested by Ottaviani and Cavazzana (1940), since they do not take up vital dyes (Taxi, 1952, 1960). Apparently, the issue has given rise to much debate long before the era of electron microscopy. With the advent of electron microscopy, Brettschneider (1962) and Suzuki (1963) considered these cells as Schwann cells. Other authors, more recently, argued that these cells are connective tissue cells on the basis of evidence from the small intestine of rabbit (Richardson, 1958), amphibians (Rogers and Burnstock, 1966), and guinea pig (Gabella, 1972). However, in the latter case, these cells would have a number of features that are atypical of connective tissue cells in that they are endowed with a well-developed smooth endoplasmic reticulum and with thin filaments inside the cell projections. In addition, the above-mentioned authors also stated that these cells are always devoid of a basal lamina, contact each other over extended areas and also come into close contact with smooth muscle cells, without any interposed basal lamina. Imaizumi and Hama (1969) described similar cells in the alimentary canal of birds and also adhered to the hypothesis that they are smooth muscle tissue, but failed to draw a definite conclusion.

It can be concluded from the above findings that the nature of interstitial cells of Cajal is totally obscure. Concerning the function of these cells, in 1925, Tiegs already postulated a role in originating, propagating and coordinating rhythmic intestinal contractile activity (myogenic rhythm, Prosser and Bortoff, 1968) but, to our knowledge, this hypothesis has not been further considered.

Interstitial cells of Cajal have not yet been described in humans by electron microscopy and our knowledge still largely corresponds to the insights provided by the studies of Cajal. These cells are not mentioned either in reports on biopsies from subjects with normal oesophageal motility and from patients with achalasia (Harman et al., 1962; Cassella et al., 1965).

Since our objective was to investigate the ultrastructural modifications of the esophagus in achalasia, we first tried to ascertain the normal ultrastructure of the oesophageal smooth muscle coat. For obvious ethical reasons, samples from subjects with a normal motility pattern could not be obtained. Instead, we analyzed samples taken from the margins of extramucosal Heller myotomies for oesophageal dyskinesia or from surgical specimens of gastric or oesophageal carcinoma. Since myotomy extends to the anterior wall of the stomach we included gastric muscle coat in our study.

## Materials and methods

Small samples of muscle coat were taken from the margins of both ends of Heller myotomies, from four subjects suffering from hypertonic dyskinesia of the lowest oesophageal one-third (one 39-years-old male), a hypertensive lower oesophageal sphincter (one 58-years-old male and one 62-years-old female) and diffuse oesophageal spasm (one 45-years-old male). Samples were taken from the esophagus, 4–5 cm above the gastro-oesophageal junction, and from the stomach, 4–5 cm below that junction. Muscle coat samples were taken from corresponding locations of patients operated on for oesophageal or gastric carcinoma (one 55-years-old male and one 60-years-old female with oesophageal carcinoma and four males, aged 45, 51, 64, and 65 years, with gastric carcinoma).

Specimens were fixed in phosphate-buffered OsO_4_ (Millonig, 1961) and embedded in Epon 812 (Luft, 1961). Sections obtained with Porter-Blum and LKB ultramicrotomes were stained with saturated aqueous solutions of uranyl acetate and lead citrate (Venable and Coggeshall, 1965) and observed with a Siemens Elmiskop Ia electron microscope.

## Results

### Smooth muscle cells

In the stomach and the esophagus of all subjects, smooth muscle cells formed small bundles that were separated from each other by loose connective tissue septa containing blood vessels and nerves as well as fibroblasts, fibrocytes, and a few macrophages and mast cells.

**Figure 1:**
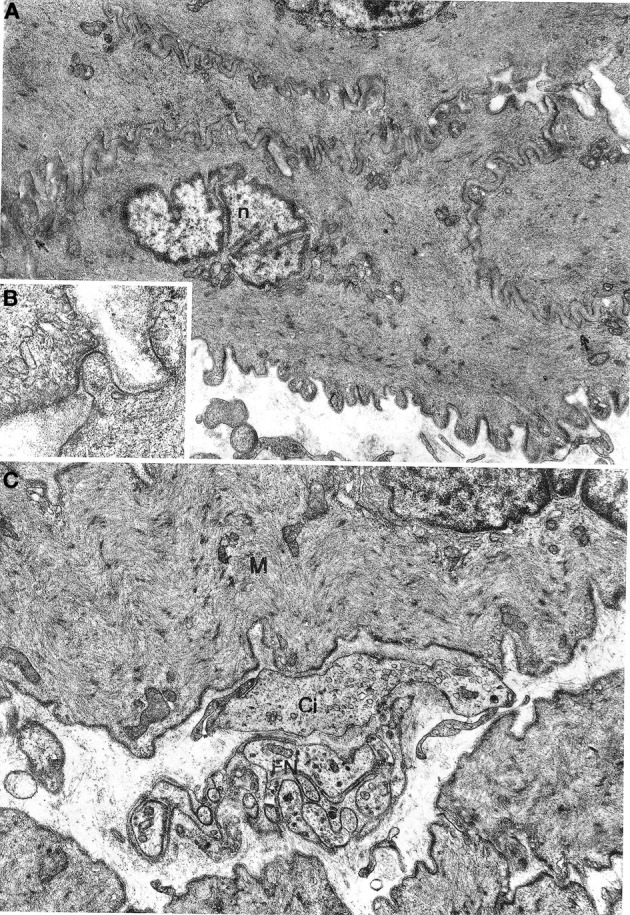
**(A,B)** Gastric muscle coat. A typical smooth muscle cells is shown in **(A)** n: nucleus. Arrows point to contact areas between adjoining cells with no basal lamina interposed. ×7800. A detail of a contact area is shown in **(B)** ×21,500. **(C)** Oesophageal muscle coat; FN, nerve bundle with five or six axons enveloped by a Schwann cell; Ci, interstitial cell; M, smooth muscle cell. ×11,400.

Smooth muscle cells (Figure 1A) were large, spindle-shaped cells with a central, ovoid nucleus; the nucleus had a wavy or deeply indented profile, presumably as a result of the contractile state of the cell. Organelles were concentrated at the cell poles: a small Golgi apparatus, mitochondria, a few ribosomes, and a small endoplasmic reticulum; a few granular vesicles and cisternae were recognized in some cells. Mitochondria were small, roundish or elongated, with usually a clear matrix and short cristae. The cytoplasm was almost entirely filled with thin filaments that were assembled into bundles and along which dense bodies, i.e., contraction spindles, were discerned. The filaments inserted into the plasma membrane in electron-dense bands. Many pinocytotic vesicles were present on the plasma membrane between dense bands. Each muscle cell was enveloped by a basal lamina that strictly followed the cell profile. Adjacent muscle cells came into close contact with each other in restricted areas where the basal lamina was interrupted. Characteristically, short projections of one cell contacted similar projections of a neighboring cell or adopted a peg-and-socket relationship to the latter cell; these cell projections were pointed or foot-like (Figure 1B). Areas of close intercellular contact were wider and often straight at the periphery of muscle cell bundles; in these cases the basal lamina appeared markedly uneven.

### Nerve fibers and nerve endings

**Figure 2:**
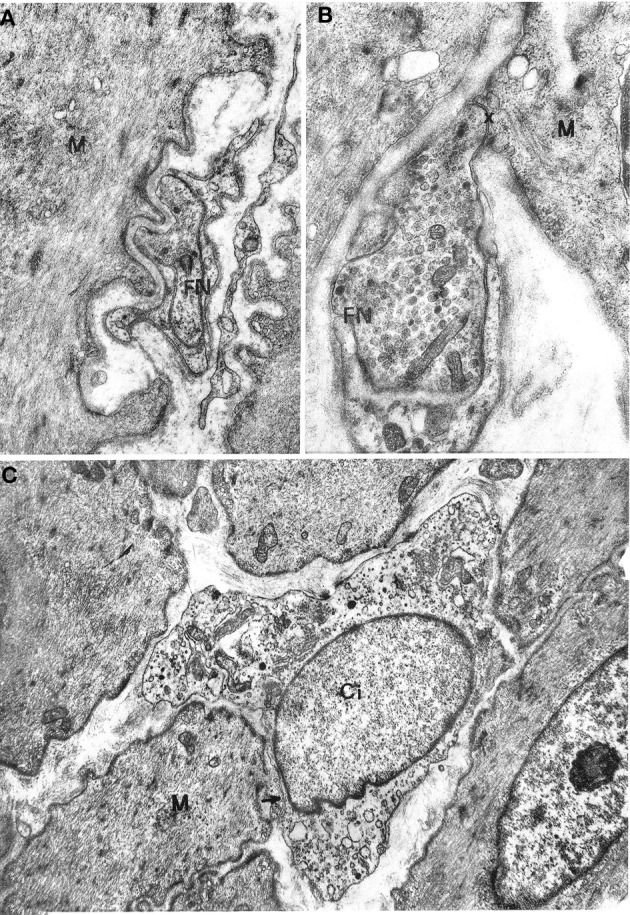
**(A,B)** Gastric muscle coat. A nerve ending (FN) is shown in **(A)** at a distance of about 1000 Å from a muscle cell (M) with a single basal lamina interposed; ×17000. A close neuromuscular junction (asterisk) between a nerve fiber (FN) and a muscle cell (M), with no basal lamina interposed, is shown in **(B)** ×19,500. **(C)** Oesophageal muscle coat. Ci: interstitial cell with cytoplasm rich in smooth endoplasmic reticulum. The cell virtually lacks a basal lamina. The arrow points to a close contact with a muscle cell (M). ×8600.

No differences between oesophageal and gastric muscle were found in this respect. Nerve bundles were usually thin, with a single Schwann cell containing 4–5 axons (Figure 1C); were typically located at the periphery of the muscle cell bundles and entered the latter rarely as branches of one to two axons. Axons expanded into multiple varicosities that were rich in mitochondria and synaptic vesicles, at a distance of approx. 1500–2000 Å from the muscle cells (Figure 2A); close junction-like contacts between nerve endings and muscle cells were rarely observed (Figure 2B). Some nerve fibers had varicosities containing large, granular vesicles, while others contained small electron-transparent vesicles; both types of nerve fibers might be involved in the rare junction-like contacts with muscle cells as described above.

### Interstitial cells of Cajal

**Figure 3:**
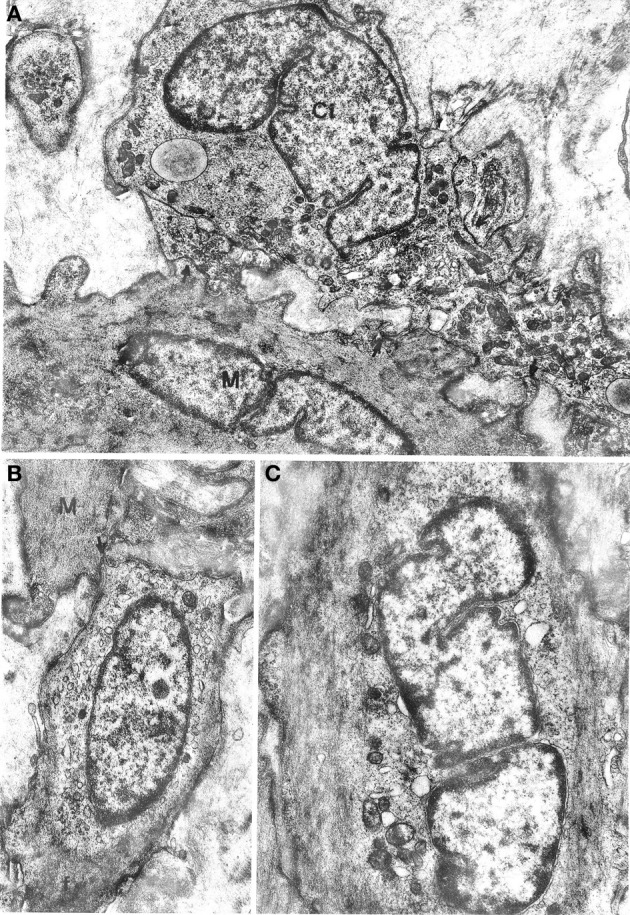
**(A)** Oesophageal muscle coat. Ci: interstitial cell rich in mitochondria and smooth endoplasmic reticulum and with a well-developed Golgi apparatus at a nuclear pole. Arrows point to close contacts with neighboring interstitial cells and with a muscle cell (M). A basal lamina surrounds the cell almost entirely. ×9400. **(B,C)** Gastric muscle coat. An interstitial cell with thin filaments (f) at the cell periphery is shown in **(B)**. The arrow points to a close contact with a muscle cell (M). ×11,400. An interstitial cell with few organelles near the nucleus and many filaments parallel to the cell major axis is shown in **(C)**. Many pinocytotic vesicles are located along the plasma membrane. ×12,800.

**Figure 4:**
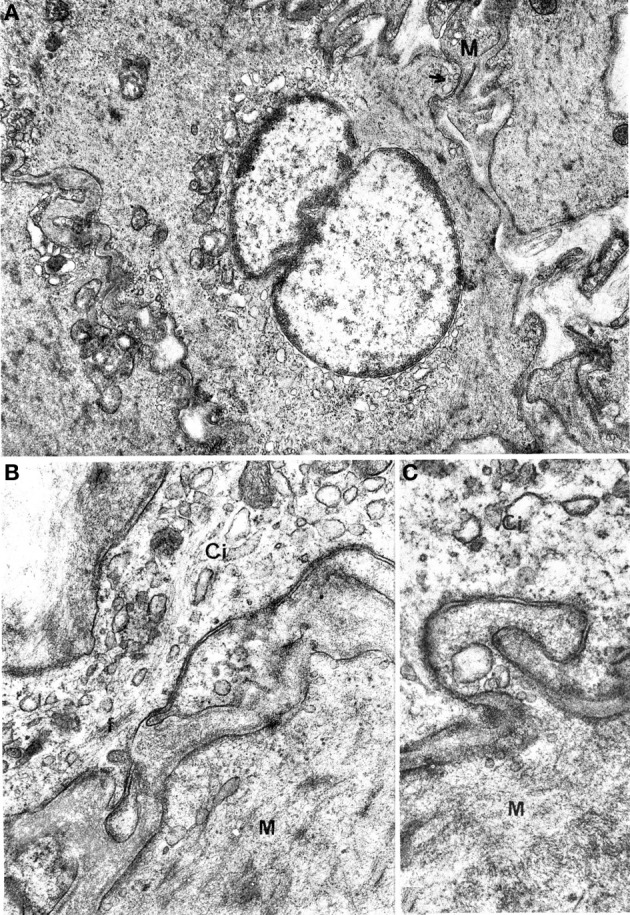
**Gastric muscle coat. (A)** A cell that is highly similar to a typical muscle cell but with a well-developed smooth endoplasmic reticulum near the nucleus. The arrow points to a close contact with a muscle cell (M). ×13,500. **(B)** A process of an interstitial cell (Ci) that is rich in vesicles of smooth endoplasmic reticulum and contains a bundle of filaments (f) parallel to the cell major axis. M: muscle cell. ×21,500. **(C)** Detail of a contact between an interstitial cell (Ci) and a muscle cell (M), without interposition of a basal lamina; ×21,500.

All oesophageal and gastric samples contained cells that we designate as interstitial cells of Cajal since they appeared to match the literature description for such cells. These cells had a variable, most often large size and irregular profile, with a slim, elongated cell body, and several cell processes of variable length (Figures 2C, 3). They were relatively few in comparison with muscle cells and were most frequently found at the periphery of muscle cell bundles. However, our observations differed in several details from those reported in the literature. The cells did not show a uniform morphology but there was no evidence of clearly distinct cell types. The nucleus was always ovoid, sometimes with an indented profile (Figures 2C, 3). The plasma membrane showed few pinocytotic vesicles, was bordered by a basal lamina with many interruptions and came into close contact with neighboring interstitial and muscle cells. The contact areas were either smooth-contoured or displayed small foot-like projections which sometimes inflected the surface of a neighboring cell in a peg-and-socket fashion. Contact areas always lacked a basal lamina (Figures 3A, 4B,C). The cytoplasmic structure of the cells was specifically polymorphic, depending on the distribution of the mitochondria and the Golgi apparatus, the development of the endoplasmic reticulum and the number of filaments. Some cells had a very well-developed endoplasmic reticulum that was made up of smooth vesicles and tubules (Figures 2C, 3A) and was sometimes concentrated around the nucleus (Figure 3B), whereas other cells had a poorly developed reticulum and quite abundant microfilaments. The latter structures, which appeared as bundles of variable thickness and were usually located at the cell periphery, ran parallel to each other and inserted into the plasma membrane in areas with an electron-dense band that were devoid of pinocytotic vesicles (Figure 3C). Some cells had a strictly perinuclear reticulum and contained filaments resembling myofilaments that filled almost the entire cytoplasm (Figure 4A); these cells could be distinguished from typical muscle cells because of extensive contacts with neighboring cells, a relative abundance of smooth endoplasmic reticulum and the presence of mitochondria and a Golgi apparatus that were not necessarily located close to the nuclear poles.

**Figure 5:**
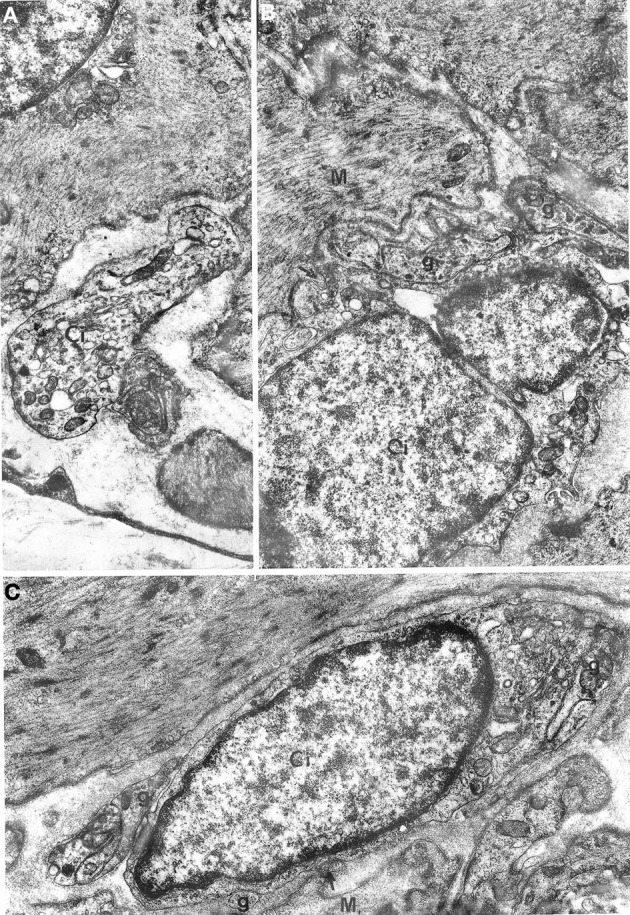
**Oesophageal muscle coat.** Relations between nerve fibers and interstitial cells are shown. **(A)** g: junctions between interstitial cells (Ci) and nerve fibers; arrows in **(B,C)** point to close contacts between interstitial and muscle (M) cells. A: ×5700. B: ×14,300. C: 13,700.

Interestingly, nerve endings were abundantly observed in the neighborhood of the interstitial cells. Many nerve bundles with synaptic vesicle-rich varicosities could be seen in almost all sections including interstitial cells; the nerve endings were in close contact with the interstitial cells (Figure 5), resulting in a markedly higher number of junctions between nerve varicosities and interstitial cells without any basal lamina interposed compared to muscle cells. This rich innervation pattern was a specific feature of interstitial cells.

## Discussion

Our observations pertaining to oesophageal smooth muscle cells corresponds to what is known from the literature for humans (Harman et al., 1962; Cassella et al., 1965). The present findings of the gastric muscle coat of humans are in keeping with reports on rat stomach (Pellegrini, 1967) and smooth muscle cells in other sites.

Our findings about innervation patterns are identical to reports on the muscle coat of the digestive system of several animal species. The innervation was composed of small nerve bundles including a few axons enveloped by one Schwann cell that were most often located at the periphery of muscle cell bundles and sometimes penetrated these latter bundles with minor branches. Some axons (cholinergic) contained small agranular vesicles, while others (possibly purinergic) contained large vesicles with an electron-dense content. Neuromuscular junctions were rare and nerve fibers were associated with muscle cells through multiple varicosities that were rich in mitochondria and synaptic vesicles, which were usually observed at a distance of more than 1000 Å from the smooth muscle cells. As such, the large distance to be covered by neurotransmitters in this atypical neuromuscular junction appears to be compensated for by a large surface area for neurotransmitter release (Bennett and Rogers, 1967; Gabella, 1972). The rare, close neuromuscular junctions were similar to those described in other organs, such as vas deferens (Richardson, 1962; Merrillees et al., 1963; Lane and Rhodin, 1964; Taxi, 1964; Yamauchi and Burnstock, 1969a,b; Faussone Pellegrini, 1972), stomach (Faussone Pellegrini, 1972), intestine (Taxi, 1961, 1964; Thaemert, 1963, 1966; Lane and Rhodin, 1964; Gabella, 1967a,b, 1972; Faussone Pellegrini, 1972), and iris (Nilsson, 1964; Richardson, 1964) of several animal species; the low incidence of these junctions is typical of the digestive system. Baumgarten et al. (1971) proposed that innervation through atypical or distant junctions, which they found in the proximal part of the excretory ducts of human testis, would be specifically adapted to modulate myogenic activity, while innervation by means of true, close junctions between nerve fibers and muscle cells would be adapted to a phasic, intense stimulation, with an “all or none” response, as observed by the same authors in the tail of epididymis and in the vas deferens.

Special attention should be paid to the cells which in this paper have been identified as those described by Cajal and therefore have been designated as “interstitial cells of Cajal,” as other authors have done before (Richardson, 1958; Brettschneider, 1962; Suzuki, 1963; Rogers and Burnstock, 1966; Imaizumi and Hama, 1969; Gabella, 1972). A first, main question pertains to the nature of these cells. The results of this study do not support the currently prevailing hypothesis that they are connective tissue cells. Important in this respect are the following specific features of these cells: (1) the presence of a basal lamina, albeit interrupted; (2) the almost complete absence of granular endoplasmic reticulum; (3) the presence of a well-developed smooth endoplasmic reticulum; (4) the presence of filaments similar to myofilaments in size and relationship to each other and to the plasma membrane; (5) the specific/surprising relationships of these cells to each other and, most importantly, to muscle cells; (6) the presence of junctions with nerve endings. All this evidence indicated that these cells cannot be regarded as fibroblasts and instead suggests that they constitute a special type of muscle cells.

This interpretation is supported by the existence of several types of interstitial cells, ranging from poorly differentiated cells with very few filaments, to highly differentiated cells that show almost all features of typical muscle cells but differ from the latter because of the number and extension of neuromuscular junctions, the localization of presumptive myofilaments to the peripheral cytoplasm and the abundant presence of mitochondria and smooth endoplasmic reticulum. Atypical smooth muscle cells were also described in other regions and are similar, but not identical to the cells described here (Gosling and Dixon, 1970, 1971, 1972; Baumgarten et al., 1971).

These findings would explain the nature of these cells, which initially were interpreted as nerve tissue cells. This hypothesis was subsequently verified but not confirmed in later studies; therefore, the hypothesis that these cells are connective tissue cells has been considered as the simplest alternative (Ottaviani and Cavazzana, 1940; Knoche, 1952; Taxi, 1952, 1960; Weber, 1952). Authors performing electron microscopic studies were somewhat biased by the fact that these cells are difficult to find in ultrathin sections and by the two prevailing hypotheses based on light microscopy, i.e., that these cells belonged to either nerve tissue (neurons or glia) or connective tissue. A smooth muscle nature has never been considered a hypothesis to date. The evidence provided here leads us to conclude that interstitial cells are specialized elements of smooth muscle tissue. It should be pointed out in this respect that it is doubtful that the cells we are dealing with are identical to those designated as interstitial cells by Cajal. It is possible that confusion arose when Cajal described these cells in 1889 and a few years later Kölliker (1894) questioned Cajal's interpretation of their nature; possibly different cell types might have been grouped under the same name; as such, intramural nerve cells and connective tissue cells might have been described as one single cell type. It cannot be excluded that the various techniques applied by Cajal led him to confound connective tissue cells, impregnated with silver following the Golgi method, with nerve cells stained intravitally with methylene blue. It is doubtful that these methods allow one to label cells which often, if not always, have features of smooth muscle cells. However, although the “interstitial cells” identified by electron microscopy may not correspond with Cajal's interstitial cells we decided to maintain the term “interstitial cells of Cajal” because of the position of these cells within smooth muscle tissue.

Two hypotheses can be put forward as to the function of interstitial cells: (1) they may be cells that are in the process of differentiating into smooth muscle cells; (2) they may be implicated in originating, conducing and coordinating the rhythmic activity which sets the pace for peristalsis in the alimentary canal. We would not support the first hypothesis since interstitial cells do not resemble embryonic myoblasts (Pellegrini, 1967, 1968c; Yamauchi and Burnstock, 1969a,b), not even the cells with very few filaments. The principal difference between these cells and embryonic myoblasts lies in the poor development of rough endoplasmic reticulum in interstitial cells compared to embryonic myoblasts. Furthermore, protracted histogenesis of the smooth muscle tissue in the organs studied here seems improbable considering the patients' age and the lack of signs of catabolism in typical muscle cells, which, if present, might be indicative of cell renewal starting from undifferentiated cells. In addition, even the cells with very few contractile filaments showed a highly specific organelle content and had a specific relationship to other interstitial cells and to typical smooth muscle cells. This morphological evidence is in disagreement with the hypothesis of interstitial cells as immature cells undergoing differentiation into smooth muscle cells.

According to the second hypothesis, which was proposed by Tiegs in 1925 and subsequently ignored, interstitial cells of Cajal might be implicated in originating, conducting and coordinating the rhythmic contractions of the alimentary canal (Holman, 1968) that have a physiological counterpart in the pacemaker-like potentials, and that are characterized by wide pre-potentials that start immediately after the falling phase of the preceding action potential. Several studies have shown that there are different patterns of membrane potential, from typical pacemaker cell patterns to patterns typical of “driven” cells (Bennett et al., 1966; Golenhofen, 1976; Prosser et al., 1976). This variety might correspond to the existence of several cell types with different contractile capacity.

The low degree of differentiation of interstitial cells as contractile elements might be linked to self-excitation, as in the myocardium (Viragh and Challice, 1973), where the specific tissue involved in generating and conducting impulses is composed of cells that are less differentiated for contraction than common myocardiocytes. Cells with few myofilaments and abundant smooth endoplasmic reticulum were encountered in the upper urinary tract (Gosling and Dixon, 1970, 1971) and in the first tract of the excretory ducts of human testis (Baumgarten et al., 1971), which are known to harbor of autonomously excitable smooth muscle tissue (Bakuntz and Vantsian, 1976). The cells found in these sites differ from those reported here, which had a well-developed rough endoplasmic reticulum and abundant glycogen. Despite minor differences in structure, we suggest that all these cells share a common function since they have similar relationships to nerve fibers, i.e., they are both characterized by multiple varicosities at a distance from the cell surface rather than by true junctions. Baumgarten et al. (1971) interpreted this fact as indicative of modulation of spontaneous muscular activity by nerve fibers rather than of strict control of motility as in skeletal and other smooth muscles. This functional hypothesis of interstitial cells is supported by their position at the periphery of smooth muscle cell bundles and their close relationships to smooth muscle cells.

In summary, the available data allow us to exclude the possibility that interstitial cells belong to connective or nerve tissues and instead permit us to put forward the hypothesis that they are specialized muscle cells which initiate myogenic contractile processes and drive typical smooth muscle cells, thus behaving as pacemaker cells.

Obviously, the available evidence only allows us to formulate such a hypothesis, which needs to be further substantiated in future studies. Important in this respect would be the finding of interstitial cells in the esophagus where, to the best of our knowledge, spontaneous contractile activity has never been demonstrated (Prosser and Bortoff, 1968). Further insight might be gained from studies dealing with the body of the esophagus, because the simplest way to confirm our hypothesis would be to find proof that the muscle coat of the esophago-gastric junction serves as an extension of cardiac gastric muscle wall, where spontaneous contractile activity has been demonstrated (see Holman, 1968). While awaiting further investigation, the interpretation of interstitial cells as pacemakers of the gastrointestinal tract may open up new research perspectives for the histophysiology of smooth muscle tissue.

## Note added in proof

While this paper was in the press, M. Yamamoto published a report on interstitial cells of the mouse and bat (Electron microscopic studies of the innervation of the smooth muscle and the interstitial cells of Cajal in the small intestine of the mouse and bat. Arch. Histol. Jap. 40, 171, 1977). This author also considered such cells as members of the smooth muscle tissue on the basis of their ultrastructure and interpreted them as undifferentiated or immature cells connecting autonomic innervation to smooth muscle tissue.

### Conflict of interest statement

The authors declare that the research was conducted in the absence of any commercial or financial relationships that could be construed as a potential conflict of interest.
